# Ruvbl2 Suppresses Cardiomyocyte Proliferation During Zebrafish Heart Development and Regeneration

**DOI:** 10.3389/fcell.2022.800594

**Published:** 2022-02-01

**Authors:** Michka Sharpe, Juan Manuel González-Rosa, Felicia Wranitz, Spencer Jeffrey, Katherine Copenhaver, C. Geoffrey Burns, Caroline E. Burns

**Affiliations:** ^1^ Division of Basic and Translational Cardiovascular Research, Department of Cardiology, Boston Children’s Hospital, Boston, MA, United States; ^2^ Cardiovascular Research Center, Massachusetts General Hospital, Charlestown, MA, United States; ^3^ Harvard Medical School, Boston, MA, United States; ^4^ Harvard Stem Cell Institute, Cambridge, MA, United States

**Keywords:** cardiomyocyte proliferation, heart development, heart regeneration, reptin/RUVBL2, zebrafish, epigenetics, chromatin remodeling

## Abstract

Cardiomyocyte proliferation is an important source of new myocardium during heart development and regeneration. Consequently, mutations in drivers of cardiomyocyte proliferation cause congenital heart disease, and infarcted human hearts scar because cardiomyocytes exit the cell cycle postnatally. To boost cardiomyocyte proliferation in either setting, critical regulators must be identified. Through an ENU screen in zebrafish, the *liebeskummer* (*lik*) mutant was isolated and described as having elevated cardiomyocyte numbers during embryogenesis. The *lik* mutation results in a three amino acid insertion into Ruvbl2, a highly conserved ATPase. Because both gain- and loss-of-function properties have been described for *ruvbl2* ^*lik*^, it remains unclear whether Ruvbl2 positively or negatively regulates cardiomyocyte proliferation. Here, we demonstrate that Ruvbl2 is a suppressor of cardiomyocyte proliferation during zebrafish heart development and regeneration. First, we confirmed speculation that augmented cardiomyocyte numbers in *ruvbl2* ^*lik/lik*^ hearts arise by hyperproliferation. To characterize bona fide *ruvbl2* null animals, we created a *ruvbl2* locus deletion allele (*ruvbl2* ^*Δ*^). Like *ruvbl2* ^*lik/lik*^ mutants, *ruvbl2* ^Δ/Δ^ and compound heterozygote *ruvbl2* ^*lik/Δ*^ animals display ventricular hyperplasia, demonstrating that *lik* is a loss of function allele and that *ruvbl2* represses cardiomyocyte proliferation. This activity is autonomous because constitutive myocardial overexpression of Ruvbl2 is sufficient to suppress cardiomyocyte proliferation in control hearts and rescue the hyperproliferation observed in *ruvbl2* ^Δ/Δ^ mutant hearts. Lastly, heat-shock inducible overexpression of Ruvbl2 suppresses cardiomyocyte proliferation during heart regeneration and leads to scarring. Together, our data demonstrate that Ruvbl2 functions autonomously as a suppressor of cardiomyocyte proliferation during both zebrafish heart development and adult heart regeneration.

## Introduction

The mammalian heart forms through sequential waves of cardiomyocyte differentiation from specialized cardiac progenitor cells (Leone et al., 2015). Subsequently, cardiomyocytes proliferate to sustain heart growth until the first postnatal week of life when they exit the cell cycle and enlarge by hypertrophy. This relatively abrupt transition correlates with a loss of regenerative capacity (González-Rosa et al., 2017; Yin et al., 2021). Much like the mammalian heart, the zebrafish heart forms through successive waves of cardiac progenitor cell differentiation that is complete by 48 h post-fertilization (hpf) (de Pater et al., 2009; Hami et al., 2011; Lazic and Scott, 2011; Zhou et al., 2011). Subsequently, the heart grows by cardiomyocyte proliferation (Choi et al., 2013). Unlike mammalian cardiomyocytes, zebrafish cardiomyocytes remain proliferative throughout life. In fact, the zebrafish heart completely regenerates lost or damaged myocardium during adulthood through robust cardiomyocyte proliferation (Pronobis and Poss, 2020). As such, the zebrafish serves as an excellent model for genetic dissection of cardiomyocyte proliferation, which could offer new therapeutic inroads for treating congenital heart disease or myocardial infarction.

Through a forward genetic screen, a recessive mutation called *liebeskummer* (*lik*) was recovered that affects heart growth during zebrafish embryogenesis (Rottbauer et al., 2002). Although *lik* mutant hearts appear qualitatively normal at 48 hpf based on whole mount *in situ* hybridization for the myocardial-specific transcript *cardiac myosin light chain 2* (*cmlc2*; also known as *myl7*), they appear enlarged by 72 hpf. Quantitative assessments were performed by counting nuclei in serial histological sections where an increase in cardiomyocyte numbers was observed in 72 hpf *lik* mutant ventricles. Although this increase was attributed to cardiomyocyte hyperplasia based on the timing of phenotype emergence, proliferation was never formally assessed.

The *lik* mutation was cloned and localized to the locus encoding Ruvbl2 (also called Reptin) (Rottbauer et al., 2002), a highly conserved ATPase in the AAA+ superfamily that functions in a wide array of biological processes including transcriptional repression and chromatin remodeling (Osaki et al., 2013). The protein structure of Ruvbl2 contains three domains including an N-terminal ATPase with Walker A and B binding motifs (Domain I), an alpha-helical domain (Domain II), and a winged helix domain predicted to bind DNA (Domain III) (Rottbauer et al., 2002). The *lik* mutation creates a novel splice acceptor site that results in a wildtype protein with 3 additional amino acids inserted into the presumptive DNA binding domain.

The functional consequence of these additional residues remains unclear due to the opposing activities of Ruvbl2^lik^ under different experimental conditions. In support of a gain-of-function protein, Ruvbl2^lik^ showed heightened ATPase activity in an *in vitro* biochemical assay, the ability to expand cardiomyocyte clones when overexpressed in mosaic zebrafish hearts, and enhanced transcriptional repression in luciferase reporter assays (Rottbauer et al., 2002). Conceptually, an increase in Ruvbl2^lik^ activity that augments cardiomyocyte proliferation is consistent with the established oncogenic role of Ruvbl2 in a variety of cancers where it is significantly overexpressed (Mao and Houry, 2017).

By contrast, genetic experiments in yeast support a loss-of-function activity that is consistent with the recessive nature of the *lik* allele in zebrafish. Specifically, yeast lacking the Ruvbl2 ortholog, RuvB (ΔyRuvB), fail to grow (Rottbauer et al., 2002). This deficiency can be rescued by expression of wildtype yRuvB, but not by yRuvB containing the analogous *lik* mutation (yRuvB^lik^). This outcome demonstrates that the *lik* mutation disrupts an essential function of the RuvB protein. Lastly, biochemical purification of tagged versions of Ruvbl2 and Ruvbl2^lik^ from cell extracts revealed that the *lik* mutation disrupts normal protein-protein interactions and results in the formation of aberrant high molecular weight complexes (Rottbauer et al., 2002). Therefore, Ruvbl2^lik^ might form novel protein associations *in vivo* that confer neomorphic properties.

Because of the functional discrepancies that are associated with the *lik* allele, no concrete conclusion has been reached regarding whether endogenous Ruvbl2 promotes or suppresses cardiomyocyte proliferation in the context of developmental heart growth. Moreover, studies designed to address potential roles for Ruvbl2 in regulating cardiomyocyte proliferation during adult heart regeneration following cardiac injury have not been performed. Here, we address these gaps in our knowledge using standard proliferation assays, new genetic strains, and the adult cryoinjury regeneration model. Together, our data demonstrate that Ruvbl2 functions autonomously to repress cardiomyocyte proliferation in the context of developmental heart growth and adult heart regeneration.

## Methods

### Zebrafish Husbandry

Zebrafish were grown and maintained according to animal protocols approved by the Massachusetts General Hospital and Boston Children’s Hospital Institutional Animal Care and Use Committee (IACUC). For adult heart regeneration experiments, animals of approximately equal sex ratios aged 4–18 months were used. Adult densities were maintained at 3–4 fish per liter. Water temperatures were maintained at 28°C except during heat shock treatments. Published strains used in this study include *Tg*(*cmlc2:nls-GFP*)^*fb18*^ (González-Rosa et al., 2018), *Tg*(*fli1a:nEGFP*)^*y7*^ (Roman et al., 2002), *ruvbl2* ^*fw039k*^ *liebeskummer* (*lik*) (Rottbauer et al., 2002) and wild type (TuAB). Details regarding the creation of new strains described in this study are below with sequence information available upon request.

### Isolation of Genomic DNA From Zebrafish Fins for Genotyping

Fins were clipped from adult zebrafish and DNA extracted through alkaline lysis. Specifically, fins were placed in a 96-well plate containing 50 μL of lysis solution (2N NaOH, 0.05M EDTA) and boiled for 20 min to 1 h in a Thermocycler at 95°C. The lysis solution was neutralized with 50 μL of 1M Tris^.^HCl (pH 5.0). PCR was performed using 2 μL of the lysis solution containing genomic DNA and allele-specific primers (see below).

### Detection of the *ruvbl2* ^*lik*^ Allele

To identify the *ruvbl2* ^*lik*^ allele, primers (Forward primer, 5′-GCC​AAA​CCT​CAT​TAT​TCA​GGC​TTT​CAT​GTG​CTT​AAA​TTG​TTA​AAT​GAC​CTC​ATA​ATG​TCA​TAT​TTC​AG-3’; Reverse primer, 5′-GAA​GTT​CCC​CCT​CTG​GAC​ACT​GCA​CAA​ACT​GCG-3′) were generated that produce an amplicon of 309 bp. No fragment is produced from the wild-type allele. The PCR program used was as follows: 95°C for 5 min (1X), 95°C for 30 s, 65°C for 30 s, 72°C for 1 min (34X), 72°C for 10 min (1X).

### Generation and Detection of the *ruvbl2* Locus Deletion (Δ) Allele

Guide RNAs targeting the sequence 5′-TGA​GTT​TCC​TGC​TGA​GCC​ACC-3′ upstream of the 5′ UTR and 5′-TGA​CAA​TCA​GTG​GCT​TGA​GGG​T-3′ downstream of the 3′UTR of the ∼12 kilobase *rubvl2* locus were generated and co-injected with Cas9 protein into one-cell stage zebrafish embryos as described (Jao et al., 2013). To detect the Δ allele, primers outside of the guide RNA cut sites (forward primer, 5′-TCC​AGA​ACT​CAT​GTA​GAC​GGT-3′; reverse primer, 5′- GTG​GCT​TGA​GGG​TCA​TGA​GA-3′) were used to yield a mutant amplicon of 266 base pairs. No fragment is produced from the wild-type allele due to the length of the intervening sequence. To detect the wild-type allele, a forward primer spanning the intron4/exon5 junction (forward primer, 5′- ATG​TCC​AGG​TAT​TGC​CCA​GTC-3′) and a reverse primer in intron 6 (reverse primer, 5′- ACC​AGC​AGA​AGC​CTA​ATA​GAG​AA—3′) were used to generate an amplicon of 601 base pairs. No fragment is produced from the locus deletion allele as the target sequence is missing. The PCR program used was as follows: 94°C for 3 min (1X), 94°C for 30 s, 52°C for 40 s, 72°C for 40 s (34X), 72°C for 5 min (1X). The allele designation is *chb8*.

### Generation of the *Tg*(*cmlc2:ruvbl2*) Transgenic Line

To generate the *Tg* (*cmlc2:EGFP-P2A-ruvbl2*) transgenic line, Gibson assembly cloning was used to generate the following construct: 1) 0.9 kb of the *myl7* promoter to drive cardiomyocyte-specific expression of 2) a bicistronic cassette encoding a nuclear localized enhanced green fluorescent protein (*nlsEGFP*) and zebrafish full-length *ruvbl2* 3) separated by a viral P2A sequence (Kim et al., 2011) and followed by 4) a polyadenylation sequence. Tol2 sites flanked the entire construct to facilitate transgenesis. The official name of this line is *Tg* (*cmlc2:nlsEGFP-P2A-ruvbl2*)*,* and the allele designation is *chb9.*

### Generation of *Tg*(*hsp70l:ruvbl2*) Line

To generate the *Tg*(*hsp70l:3XHA-ruvbl2*) transgenic line, Gibson Assembly was performed to generate the following construct: 1) ∼1.5 kb of the *hsp70l* promoter (Kwan et al., 2007) was used to drive expression of 2) three copies of the HA tag fused in-frame to the 5′ end of zebrafish *ruvbl2* (3XHA-*ruvbl2*) followed by a 3) *Xenopus* β-globin 3’ UTR, and 4) bGH polyadenylation signal. To fluorescently label transgenic embryos, the EF1α promoter was cloned upstream of GFP in the opposite orientation of the heat-shock cassette. Tol2 sites flanked the entire construct. The official name of this line *is Tg* (*hsp70l:3xHA-ruvbl2*, *eef1a1l1:EGFP*), and the allele designation is *chb10*.

### Imaging

Live zebrafish embryos were imaged on a Nikon 80i Compound Microscope with Retiga 2000R high-speed charge-coupled device (CCD) camera (*QImaging*) and NIS-Elements advanced research image acquisition and analysis system (Nikon Instruments). The resulting images were focused stacked with Zerene Stacker Software (Build T201412212230). Following immunostaining, fixed zebrafish embryos were embedded in 0.9% low-melt agarose (Lonza, USA) in glass bottom dishes (MatTek) and covered with PBSTw (PBS with 0.1% Tween) prior to imaging on a Nikon Eclipse A1 confocal microscope with a 40x Nikon Plan Apo water objective. Images were analyzed with Fiji software (Schindelin et al., 2012).

### BrdU Proliferation Assay

BrdU labelling and immunostaining was performed as previously described (Jahangiri et al., 2016). Specifically, embryos were incubated in 5 mg/ml 5-Bromo-2′deoxyuridine (BrdU), 1% DMSO (Sigma-Aldrich, USA) in E3 from 48 to 72 hpf at 28°C, rinsed three times in PBST and fixed overnight in 4% paraformaldehyde. Fixed embryos were processed as described (Jahangiri et al., 2016) and immunostained with anti-MF20 and anti-BrdU (see below). Embryos were mounted and imaged by confocal microscopy. A myocardial proliferation index (MF20+; BrdU+ cell number divided by the total MF20+ cell number) was calculated for each embryo.

### RNAscope Expression Analysis

For whole mount *in situ* hybridization, 72 hpf embryos were fixed for 4 h in 4% PFA, washed with PBSTw and dehydrated in methanol at 20°C overnight. Whole mount fluorescent *in situ* hybridization was performed utilizing RNAscope (Advanced Cell Diagnostics) as previously described (Guner-Ataman et al., 2018). RNAscope riboprobes to *ruvbl2* were hybridized overnight at 40°C. Following the final labeling reaction, embryos were incubated with DAPI solution overnight at 4°C to visualize nuclei. Embryos were imaged on a Nikon Eclipse A1 confocal microscope.

For adult cardiac sections, uninjured or 7 dpi adult zebrafish hearts were dissected as described (González-Rosa et al., 2011) and fixed for 24 h in 10% neutral buffered formalin at room temperature. Fixed hearts were washed, dehydrated, and embedded in paraffin. 7 µm sections were obtained using a Leica Microtome. *In situ* hybridization of *ruvbl2* transcripts in sections was performed using the RNAscope Multiplex Fluorescent V2 kit, following the manufacturer’s instructions. The mRNA signal was detected using the Opal 570 reagent (Akoya Biosciences, 1:1,000). Following *ruvbl2* detection, sections were incubated overnight with anti-GFP (clone B-2, Santa Cruz Biotechnology, 1:200) antibodies, and nuclei were counterstained using DAPI. Slides were mounted in FluorSafe and imaged using a Zeiss LSM900 confocal microscope. To quantify *ruvbl2* expression, 2-3 ventricular sections containing the largest injury areas were imaged. *ruvbl2*+ punctate dots were counted manually using ImageJ software in defined regions (50 μm × 50 µm) located at the border of the injured area (border zone, BZ) and the remote myocardium (RM). The average number of *ruvbl2*+ dots in the BZ and RM was calculated for each animal. For statistical comparisons, all values were normalized to the average count in the remote myocardium.

### Whole Mount Immunofluorescence

Whole-mount immunostaining was performed as previously described (Abrial et al., 2017). Primary antibodies used were mouse monoclonal anti-GFP (1:50, Santa Cruz, USA), rabbit polyclonal anti-GFP (1:200, Abcam, USA), mouse monoclonal anti-myosin heavy chain (MF20; 1:50, Developmental Studies Hybridoma Bank, USA), and mouse monoclonal anti-BrdU (1:100; clone BMC9318, Roche, Sigma Aldrich, USA). Alexa conjugated antibodies (Invitrogen, USA) were used at 1:500 dilutions to reveal primary antibody signal. Nuclei were stained with DAPI (1:1,000, Life Technologies, USA).

### Zebrafish Cardiac Cryoinjury

Regeneration experiments were conducted using adult zebrafish as described (González-Rosa et al., 2011). Briefly, fish were anesthetized in tricaine, placed with their ventral side up on a slotted sponge and a small incision made to expose the ventricle. A platinum wire probe cooled in liquid nitrogen was touched to the ventricular surface until thawing was observed. After surgery, animals were returned to a tank of fresh water and revived by gently pulsing water over their gills using a plastic Pasteur pipette.

### Heat Shock Treatments

Adult heat shock treatments were performed as previously described (Zhao et al., 2014). Following ventricular cryoinjury, adult zebrafish with (experimental) or without (control) the *Tg(hsp70l:3XHA-ruvbl2)* transgene were allowed to recover overnight in the aquatics facility. On 1 dpi, animals were moved to an automated heat-shock rack where they were exposed to daily increases in temperature from 28°C to 39°C for 1 h followed by gradual decreases in temperature to 28°C. At 7 and 60 dpi animals were euthanized and their hearts dissected for histological analysis.

### Metanalysis of ruvbl2 Expression Dynamics During Heart Regeneration Using the Zebrafish Regeneration Database Online Resource

The zfRegeneration.org database (Nieto-Arellano and Sánchez-Iranzo, 2019) was used to survey *ruvbl2* expression levels in publicly available heart regeneration datasets. The “Plot fpkm values” function was selected, and *ruvbl2* levels were retrieved using the “All zebrafish datasets” option. The resulting plots were downloaded as a single .png file. Only the plots depicting heart regeneration were selected for evaluation.

### Histological Staining

Dissected hearts were fixed in 4% paraformaldehyde in phosphate buffered saline (PBS) overnight at 4°C, included in paraffin, and sectioned. Immunofluorescence on paraffin sections was performed as described (González-Rosa et al., 2011) using the following primary antibodies: rabbit anti-Mef2 (Cat#sc-313; RRID: AB_631920; Santa Cruz Biotechnology, 1:100) and mouse monoclonal anti-PCNA (Sigma-Aldrich, Cat#WH0005111M2; RRID: AB_1842895, 1:200) and Alexa conjugated secondary antibodies (Life Technologies, 1:500) were used to detect primary antibody signals. Nuclei were counterstained with DAPI (1:1,000, Life Technologies, USA) and slides were mounted in FluorSafe prior to imaging on a Nikon A1 confocal microscope. Acid fuchsin-orange G (AFOG) stain was used to detect the fibrotic tissue. Muscle, fibrin, and collagen were stained brownish orange, red, and blue, respectively.

### Quantification of Myocardial Proliferation and Scar Retention Analysis in Regenerating Hearts

Myocardial proliferation indices were quantified as previously reported (Zhao et al., 2019). Briefly, ventricular sections were immunstained with anti-Mef2 and anti-PCNA (see above). Three sections per heart containing the largest injury area were imaged by confocal microscopy. The number of Mef2+ and Mef2+; PCNA+ cells were counted manually using Fiji software in a defined region (200 μm × 424.55 μm) that included the injury area and border zone. Percentages of proliferative cardiomyocytes (Mef2+; PCNA+/Mef2+) were calculated from individual sections and averaged to establish a myocardial proliferative index for each animal. To assess fibrosis, images of AFOG-stained serial sections of whole hearts were captured as described in (González-Rosa et al., 2018). On average, ∼15 sections were analyzed per heart.

### Statistical Analysis

Samples sizes were chosen based on previous publications and indicated in each figure legend. No animal or sample that was evaluated was excluded from the analysis. The investigators were blinded to genotype when assessing cardiomyocyte proliferation and scar formation for heart regeneration experiments. All statistical values are displayed as mean ±1 standard deviation (SD). Sample sizes and *p* values are indicated in the figures or figure legends. Student’s t test was used to compare the means from two groups and the one way analysis of variants (ANOVA) was used to compare more than two groups. Statistical significance was assigned at *p* < 0.05. All statistical tests were performed using Prism8 software.

## Results

**FIGURE 1:**
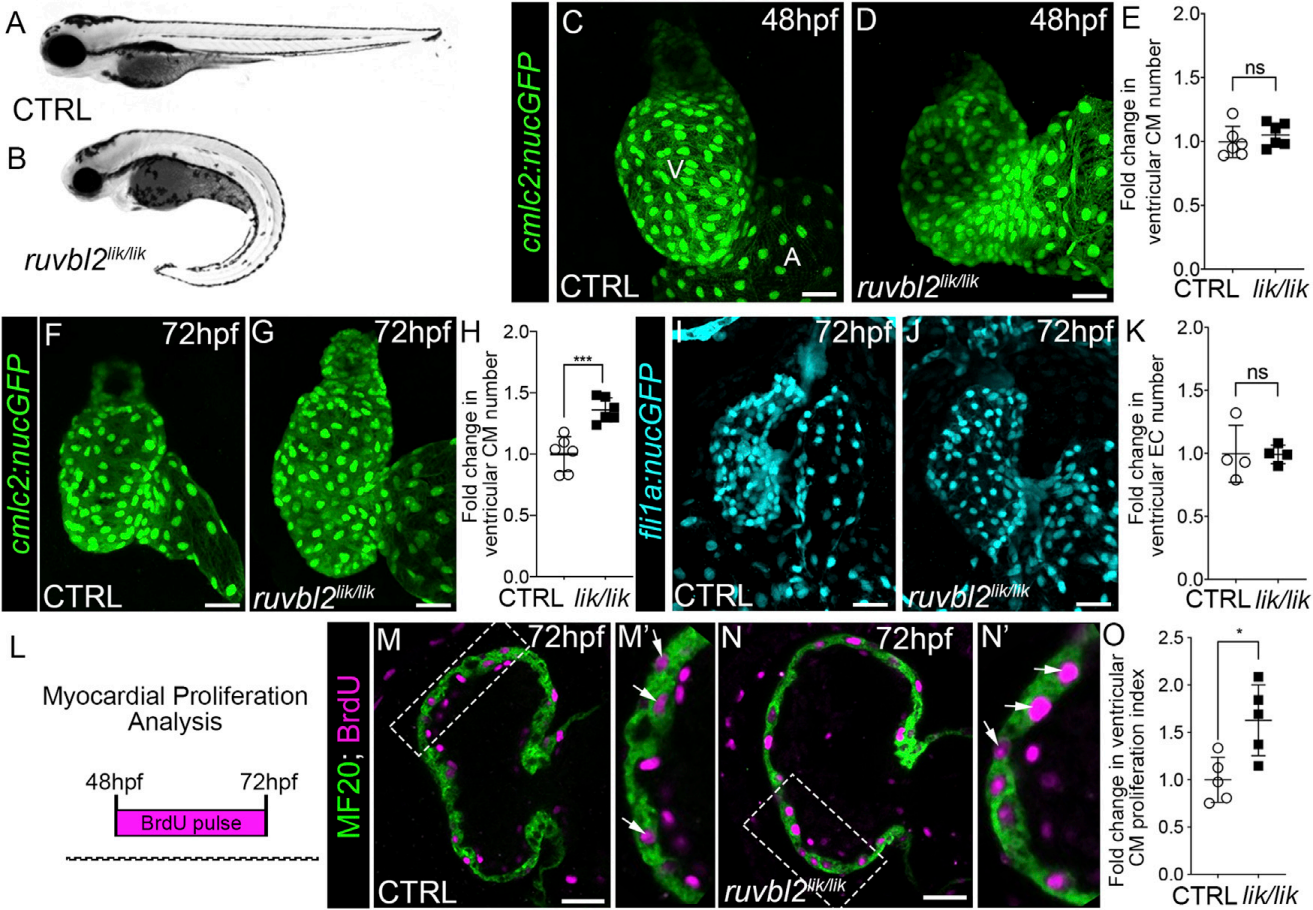
*ruvbl2* ^*lik*^ mutant hearts show increased cardiomyocyte proliferation. Brightfield images of wild-type **(A)** and *ruvbl2* ^*lik/lik*^ **(B)** embryos at 72 hpf. Anterior left, lateral view. **(C,D)** Confocal projections of fluorescent cardiomyocyte nuclei in hearts of 48 hpf control (CTRL) sibling (C; *n* = 6) and *ruvbl2* ^*lik/lik*^ (D, *n* = 6) *Tg* (*cmlc2:nucGFP*) embryos V = ventricle; A = atrium. **(E)** Quantification of fold change in ventricular cardiomyocyte number in 48 hpf CTRL and *ruvbl2* ^*lik/lik*^ hearts. Means ± s.d are shown. Not significant (ns); *p* > 0.05. **(F,G)** Confocal projections of fluorescent cardiomyocyte nuclei in hearts of 72 hpf CTRL (F; *n* = 6) and *ruvbl2* ^*lik/lik*^ (G; *n* = 6) *Tg* (*cmlc2:nucGFP*) embryos. **(H)** Quantification of fold change in ventricular cardiomyocyte number in 72 hpf CTRL and *ruvbl2* ^*lik/lik*^ hearts. Means ± s.d are shown. ***p < 0.0005. **(I,J)** Confocal projections of fluorescent endocardial nuclei in hearts of 72 hpf CTRL (I; *n* = 4) and *ruvbl2* ^*lik/lik*^ (J; *n* = 4) *Tg* (*fli1a:nucGFP*) embryos. **(K)** Quantification of fold change in ventricular endocardial cell number in 72 hpf CTRL and *ruvbl2* ^*lik/lik*^ hearts. Means ± s.d are shown. Not significant (ns); *p* > 0.05. **(L)** Schematic representing the myocardial proliferation assay by BrdU pulse between 48 and 72 hpf. **(M–N′)** Single plane confocal images of CTRL (M; *n* = 5) and *ruvbl2* ^*lik/lik*^ (N; *n* = 5) hearts double immunostained to detect cycling (BrdU+) cardiomyocytes (MF20+) at 72 hpf. Boxed regions in **(M,N)** are magnified in **(M′,N′)** with white arrows highlighting double positive cells. **(O)** Quantification of cardiomyocyte proliferation indices in CTRL and *ruvbl2* ^*lik/lik*^ ventricles. Means ± s.d are shown. *p < 0.05. Scale bars: 25 μm.

A previous report claimed that zebrafish embryos homozygous for the *ruvbl2* ^*lik*^ mutation display cardiomyocyte hyperplasia based on myocardial nuclear counts in serial paraffin sections after histological staining (Rottbauer et al., 2002). We sought to confirm and extend these findings using newer fluorescent reporter strains, BrdU labelling of proliferative cardiomyocytes, and confocal imaging techniques that were not readily available at the time of the prior study. 25% of the embryos generated from *ruvbl2* ^*lik*/+^ heterozygous incrosses displayed curved body morphologies characteristic of *ruvbl2* ^*lik/lik*^ mutants that we verified by genotyping (Figures 1A,B).

During zebrafish heart development, cardiomyocyte numbers increase within the forming ventricular chamber through progenitor cell differentiation prior to 48 hpf and by cardiomyocyte proliferation thereafter (Choi et al., 2013). To learn during which phase Ruvbl2 regulates cardiac growth, we quantified ventricular cardiomyocyte numbers at 48 and 72 hpf in control and *ruvbl2* ^*lik/lik*^ animals carrying the myocardial nuclear reporter *Tg(cmlc2:nucGFP)*. While no significant difference was observed between cohorts at 48 hpf (Figures 1C–E), a 1.36-fold increase in ventricular cardiomyocyte numbers was detected at 72 hpf (Figures 1F–H), demonstrating that Ruvbl2 functions during the proliferative phase of chamber morphogenesis. This expansion is specific to the myocardium as no significant difference in endocardial cell numbers was observed between control and *ruvbl2* ^*lik/lik*^ animals carrying the endothelial nuclear reporter *Tg(fli1a:nucGFP)* at similar developmental stages (Figures 1I–K). To formally assess cardiomyocyte proliferation, we treated control and *ruvbl2* ^*lik/lik*^ embryos with BrdU at 48 hpf and performed immunofluorescence with anti-BrdU and the muscle-specific MF20 antibody followed by DAPI staining at 72 hpf (Figure 1L). After confocal imaging, we quantified the number of BrdU+ cells within the MF20+; DAPI+ ventricular myocardium to generate a cardiomyocyte proliferation index. *Ruvbl2* ^*lik/lik*^ mutants show a 1.63-fold higher rate of proliferation compared to controls (Figures 1M–O). Together, these data confirm and extend previous observations demonstrating that Ruvbl2 functions to regulate myocardial proliferation during zebrafish ventricular chamber growth without affecting progenitor cell differentiation.

**FIGURE 2:**
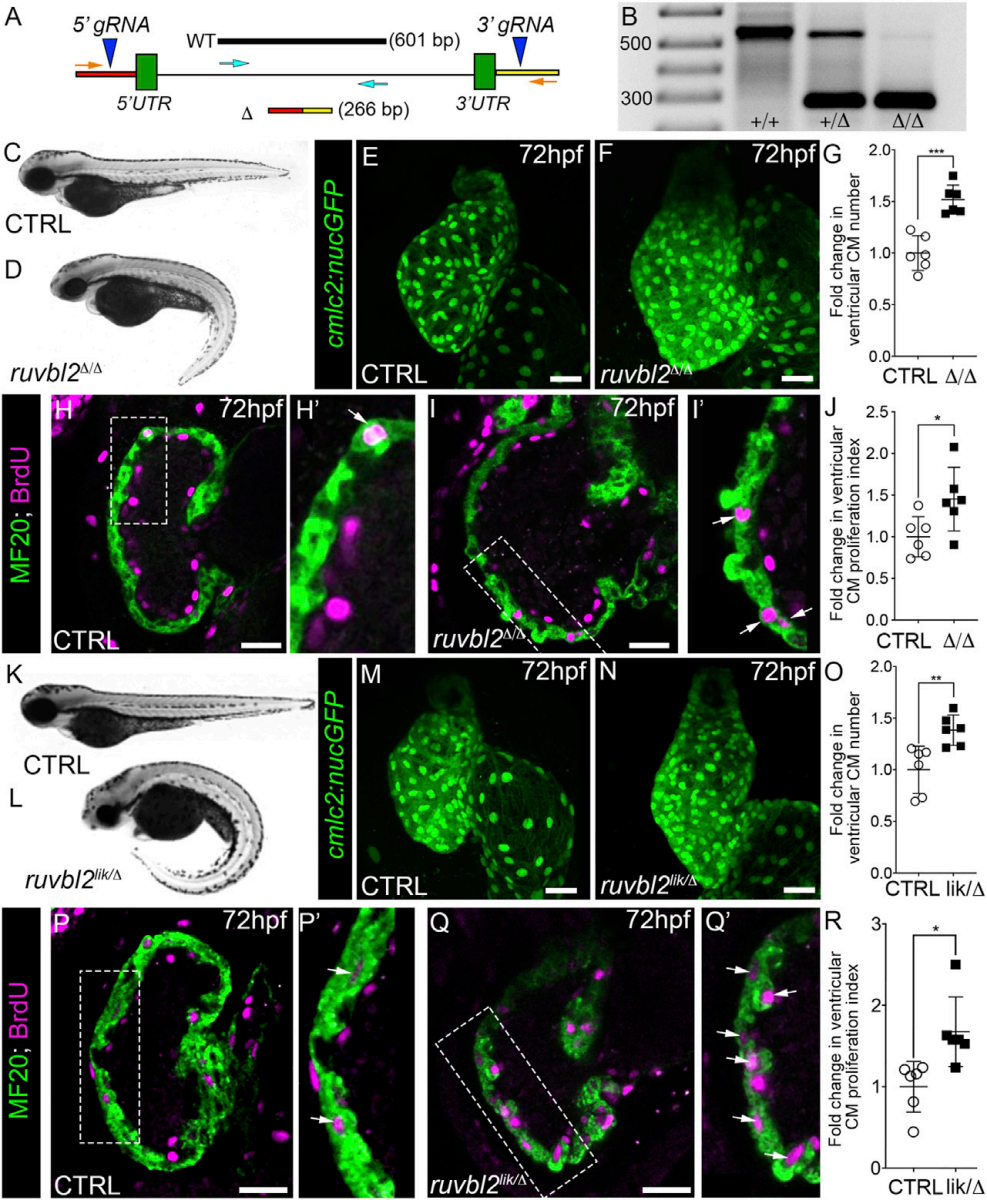
Ruvbl2 suppresses cardiomyocyte proliferation during embryonic heart growth. **(A)** Schematic of the *ruvbl2* locus deletion (*ruvbl2* ^*Δ*^) targeting strategy by CRISPR-Cas9 genome editing. gRNAs (blue arrowheads) were designed that target genomic regions outside of the *ruvbl2* 5′ and 3′ UTR. Location of PCR primers used to detect the wild-type (WT; turquoise arrows) and mutant (Δ; orange arrows) alleles. **(B)** Image of a DNA agarose gel showing amplicons for the wild-type (+) and mutant (Δ) alleles. **(C,D)** and **(K,L)** Brightfield images of wild-type **(C,K)**, *ruvbl2* ^*Δ/Δ*^ **(D)**, *ruvbl2* ^*lik/Δ*^ **(L)** embryos at 72 hpf. Anterior left, lateral view. **(E,F)** and **(M,N)** Confocal projections of fluorescent cardiomyocyte nuclei in hearts of 72 hpf CTRL (F; *n* = 6 and M; *n* = 6), *ruvbl2* ^*Δ/Δ*^ (G; *n* = 6), and *ruvbl2* ^*lik/Δ*^ (N; *n* = 6) *Tg* (*cmlc2:nucGFP*) embryos. **(G,O)** Quantification of fold change in ventricular cardiomyocyte number in 72 hpf CTRL and *ruvbl2* ^*Δ/Δ*^ **(G)** or CTRL and *ruvbl2* ^*lik/Δ*^ **(O)** hearts. Means ± s.d are shown. ***p < 0.001; **p < 0.01. **(H–I’,P–Q′)** Single plane confocal images of CTRL (H; *n* = 6 and P; *n* = 6), *ruvbl2* ^*Δ/Δ*^ (N; *n* = 6), and *ruvbl2* ^*lik/Δ*^ (Q; *n* = 6) hearts double immunostained to detect cycling (BrdU+) cardiomyocytes (MF20+) at 72 hpf. Boxed regions in **(H,I,P,Q)** are magnified in **(H′,I′,P′,Q′)** with white arrows highlighting double positive cells. **(J,R)** Quantification of cardiomyocyte proliferation indices in CTRL and *ruvbl2* ^*Δ/Δ*^ **(J)** or CTRL and *ruvbl2* ^*lik/Δ*^ **(R)** ventricles. Means ± s.d are shown. *p < 0.05. Scale bars: 25 μm.

The functional consequence of the *lik* mutation has been unclear due to the opposing activities of Ruvbl2^lik^ under different experimental conditions (see *Introduction*). As such, it has remained unknown whether Ruvbl2 promotes or suppresses cardiomyocyte proliferation during developmental heart growth. To resolve this issue, we generated a bona fide loss-of-function allele using CRISPR-Cas9 genome editing by deleting the entire endogenous locus. Specifically, guide RNAs targeting the genomic regions upstream and downstream of the *ruvbl2* 5′ and 3′ UTRs, respectively were co-injected with Cas9 protein into one-cell zebrafish embryos with the intent of releasing the intervening sequence (Figure 2A). A whole locus deletion allele (designated Δ) was isolated, confirmed by PCR genotyping (Figure 2B) and Sanger sequencing, and propagated in the F1 generation.

Breeding of *ruvbl* ^*Δ*/+^ heterozygotes resulted in roughly 25% of their progeny showing curved body morphologies similar to that of *ruvbl2* ^*lik/lik*^ mutants (Figures 2C,D). Next, we imaged hearts from control and *ruvbl2* ^*Δ/Δ*^ mutants carrying the *Tg*(*cmlc2:nucGFP*) reporter. In comparison to controls, the ventricle of *ruvbl2* ^*Δ/Δ*^ mutants appeared significantly larger (Figures 2E,F). Quantification of myocardial nuclei confirmed a 1.52-fold increase in ventricular cardiomyocyte numbers (Figures 2E–G) that is associated with a 1.45-fold increase in the proliferation index (Figures 2H–J). Together, these data demonstrate that *ruvbl2* ^*Δ/Δ*^ mutants phenocopy the ventricular hyperplasia documented in *ruvbl2* ^*lik/lik*^ mutants (Figure 1), suggesting that both alleles create loss-of-function proteins.

To test this hypothesis directly, we bred *ruvbl2* ^*+/lik*^ to *ruvbl2* ^*+/Δ*^ adults to generate compound heterozygous *ruvbl2* ^*lik/Δ*^ progeny that we analyzed for the previously described phenotypes. We found that *ruvbl2* ^*lik/Δ*^ embryos display curved body morphologies (Figures 2K,L) and have hearts with 1.38-fold more ventricular cardiomyocytes (Figures 2M–O) that are 1.66-fold more proliferative (Figures 2P–R) than controls. From these analyses, we conclude that Ruvbl2^lik^ encodes a loss-of-function protein with respect to cardiomyocyte proliferation.

**FIGURE 3:**
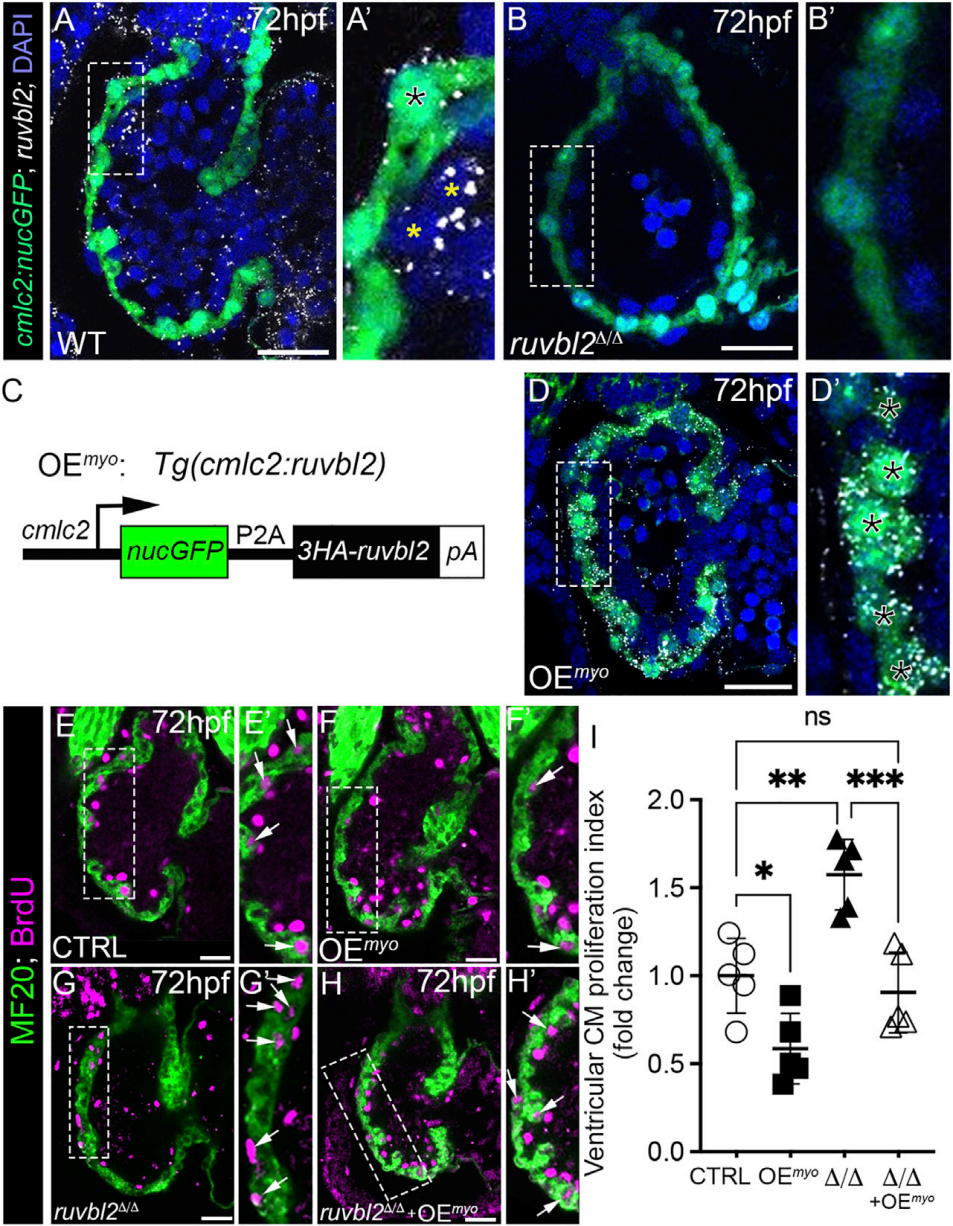
Myocardial expression of Ruvbl2 rescues cardiomyocyte hyperproliferation in *ruvbl2* ^*Δ/Δ*^ mutants. **(A–B′)** Confocal single plane optical sections of endogenous *ruvbl2* expression (white signal) relative to myocardium (green signal) processed by RNAscope fluorescent *in situ* hybridization and immunohistochemistry for GFP in wild-type (A, A′; *n* = 4) or *ruvbl2* ^*Δ/Δ*^ (B, B′; *n* = 4) *Tg* (*cmlc2:nucGFP*) embryos with DAPI nuclei counterstaining (blue signal). Boxed regions in A and B are shown at higher magnifications in A′ and B′, respectively. The black asterix in A′ denotes myocardial *ruvbl2* expression, while yellow astrices mark presumptive endocardial *ruvbl2* expression. **(C)** Schematic of the transgene generated to achieve constitutive *ruvbl2* overexpression in the myocardium (OEmyo). **(D)** Confocal single plane optical section of the *ruvbl2* expression domain (white signal) relative to myocardium (green signal) processed by RNAscope fluorescent *in situ* hybridization and immunohistochemistry for GFP in *Tg* (*cmlc2:ruvbl2*) *Tg* (*cmlc2:nucGFP*) double transgenic embryos (*n* = 4) with DAPI nuclei counterstaining (blue signal). Boxed region in D is shown at higher magnifications in D′. Black asterices in **(D′)** denote myocardial *ruvbl2* expression, which when compared to wildtype **(A,A′)** is substantially higher. **(E–H′)** Single plane confocal images of wildtype control **(E,E′)**, OEmyo **(F,F′)**, *ruvbl2* ^*Δ/Δ*^ **(G,G′)**, *ruvbl2* ^*Δ/Δ*^ with OEmyo **(H,H′)** hearts double immunostained to detect cycling (BrdU+) cardiomyocytes (MF20+) at 72 hpf. Boxed regions in **(E–H)** are magnified in **(E′–H′)** with white arrows highlighting double positive cells. Sample sizes are *n* = 6 for all groups. **(I)** Quantification of fold change in cardiomyocyte proliferation indices between cohorts. Means ± s.d are shown. ns; not significant; *p < 0.05, **p < 0.01, ***p < 0.001. Scale bars: 25 μm.

To spatially resolve the cardiac cell types expressing *ruvbl2*, we used RNAscope *in situ* hybridization to detect *ruvbl2* transcripts in 72 hpf zebrafish embryos carrying the *Tg(cmlc2:nucGFP)* myocardial reporter. In single confocal sections, *ruvbl2* transcripts co-localize with GFP in ventricular cardiomyocytes and in presumptive endocardial cells medial to the GFP signal (Figures 3A,A′). As anticipated, we failed to detect *ruvbl2* transcripts in *ruvbl2* ^*Δ/Δ*^ mutants (Figures 3B,B′), demonstrating specificity of the signal in wildtype animals.

Because *ruvbl2* is endogenously expressed in cardiomyocytes, we tested whether myocardial-specific Ruvbl2 overexpression is sufficient to inhibit cardiomyocyte proliferation in wildtype embryos and capable of rescuing the cardiomyocyte hyperplasia observed in *ruvbl2* ^*Δ/Δ*^ mutants. To this end, we generated a stable transgenic strain that constitutively expresses a nuclear-localized GFP followed by a viral P2A sequence and a 3XHA tagged-Ruvbl2 in cardiomyocytes that we termed overexpression in the myocardium (OE^*myo*^; Figure 3C). To validate the strain, we performed RNAscope as described above and found substantially more *ruvbl2* transcripts in OE^*myo*^ hearts than in control (compare Figures 3D,D′ to Figures 3A,A′). As anticipated, the increase in transcripts is restricted to the myocardium and not observed in the endocardium. Next, we compared the ventricular cardiomyocyte proliferation index between control and OE^*myo*^ hearts using the same BrdU pulse-chase assay as described previously. We found that cardiomyocytes overexpressing Ruvbl2 are 42% less proliferative than controls (Figures 3E–F′ and Figure 3I), demonstrating that Ruvbl2 is sufficient to suppress cardiomyocyte proliferation during developmental heart growth. Consistent with our prior analysis (Figures 2H–J), *ruvbl2* ^*Δ/Δ*^ mutant hearts are 1.57-fold more proliferative than controls (Figures 3E,E′,G,G′,I). However, in the presence of the OE^*myo*^ transgene, the hyperplasia observed in *ruvbl2* ^*Δ/Δ*^ mutant hearts is rescued back to control levels (Figures 3E,E′,G–I), demonstrating an autonomous function for Ruvbl2 in the myocardium for suppressing proliferation. This claim is further supported by prior studies where mosaic zebrafish ventricles showed clonal expansion of both transplanted *ruvbl2* ^*lik*^ mutant cells and cardiomyocytes constitutively overexpressing Ruvbl2^lik^ (Rottbauer et al., 2002).

**FIGURE 4:**
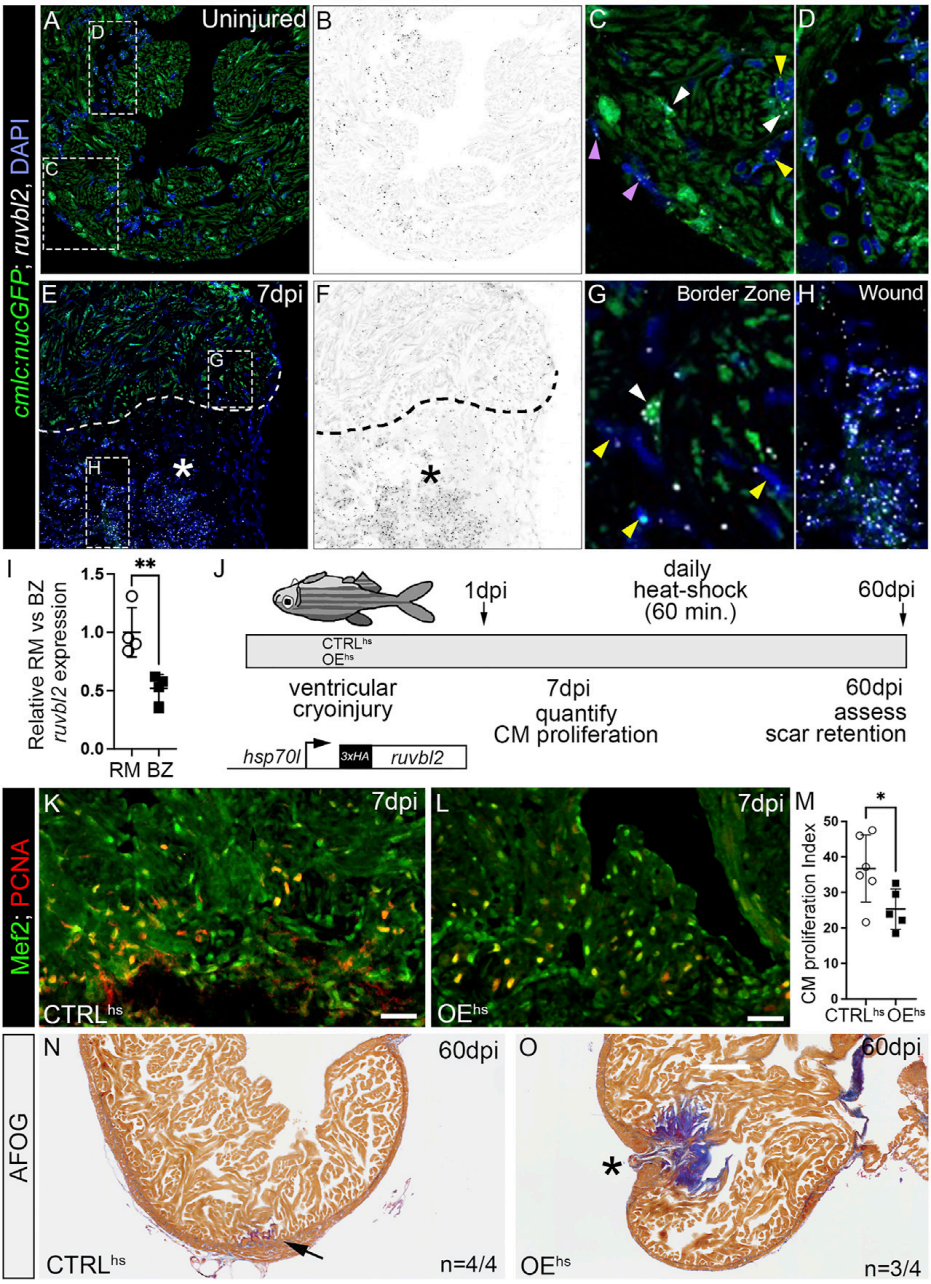
*ruvbl2* suppresses cardiomyocyte proliferation during zebrafish cardiac regeneration and leads to scar retention. **(A–H)** RNAScope *in situ* hybridization of *ruvbl2* transcripts in representative cardiac sections from uninjured or 7 dpi hearts immunostained for GFP and counterstained with DAPI. *Ruvbl2* fluorescent signals in **(A,E)** are pseudocolored to black and white in **(B,F)**. Boxed regions in A and E are shown in magnified views in **(C,D,G,H)** as indicated. **(C,G)** Purple arrowhead, epicardial cells; white arrowhead, myocardial cells; yellow arrowhead, endocardial cells. **(D)** *Ruvbl2* expression in circulating cells. **(E,F)** Dotted line shows the border between the wound (marked with an asterisk) and the spared cardiac tissue. **(I)** Quantification of relative *ruvbl2* expression between the remote myocardium (RM) and border zone (BZ). **(J)** Schematic of experimental design for regeneration experiments. Adult zebrafish carrying the *Tg* (*hsp70l:ruvbl2*) overexpression transgene were given a single heat shock per day following cryoinjury to the ventricle. Experimental endpoints were at 7 dpi and 60 dpi to assay cardiomyocyte proliferation and regeneration, respectively. **(K,L)** Sections of WT and *Tg* (*hsp70l:ruvbl2*) hearts immunostained for Mef2c and PCNA at 7 dpi. **(M)** Quantification of the cardiomyocyte proliferation index at 7 dpi. **(N,O)** Representative sections of WT and *Tg* (*hsp70l:ruvbl2*) hearts stained with acid fuchsin orange G (AFOG) at 60 dpi. Black arrow highlights regenerated wound area. Black asterisk highlights fibrotic scar tissue with open myocardial wall. 4 of 4 WT hearts regenerated well and 3 of 4 OE^hs^ hearts retained substantial scar tissue as shown. Mean ± SD are shown. *p < 0.05; **p < 0.01. Scale bars: 25 μm.

In addition to developmental heart growth, zebrafish heart regeneration during adulthood also relies on cardiomyocyte proliferation (Jopling et al., 2010; Kikuchi et al., 2010). To examine the spatiotemporal expression of *ruvbl2* in adult hearts before and after insult, we performed cryoinjury on their ventricles as previously described (González-Rosa et al., 2011; González-Rosa and Mercader, 2012). Hearts were harvested from uninjured or 7 days post-injury (dpi) animals carrying the myocardial reporter *Tg(cmlc2:nucGFP)*, sectioned, and processed by RNAscope *in situ* hybridization for *ruvbl2* transcripts followed by antibody staining for GFP. During homeostasis, adult hearts express relatively low but detectable levels of *ruvbl2* in cardiomyocytes, endocardial cells, epicardial cells (Figures 4A–C), and circulating cells (Figures 4A,B,D). On 7 dpi, we observed a qualitative increase in *ruvbl2* expression that appeared largely restricted to the wound in presumptive endocardial and immune cells (Figures 4E–H). In support of these data, we searched the Zebrafish Regeneration Database that contains published transcriptome datasets of heart regeneration (http://zfregeneration.org) (Nieto-Arellano and Sánchez-Iranzo, 2019) and found a similar pattern of expression with low numbers of *ruvbl2* reads in uninjured hearts that increase following injury (Supplementary Figure S1). In cardiac sections, *ruvbl2* expression is lower in border zone (BZ) cardiomyocytes compared to remote myocardium (RM) (Figure 4I), demonstrating that there are less *ruvbl2* transcripts in the region where cardiomyocytes are most proliferative.

To learn whether Ruvbl2 is sufficient to suppress cardiomyocyte proliferation in the context of heart regeneration, we created a new inducible transgenic line where the *heat-shock 70l* promoter drives expression of a 3xHA tagged version of *ruvbl2* that we termed overexpression by heat-shock (OE^hs^; Figure 4J). We grew non-transgenic control and OE^hs^ siblings to adulthood, performed cryoinjury on their ventricles, and allowed them to recover for 1 day. We then placed cryoinjured fish of like genotype on an automated heat-shock rack that increased the water temperature to 37°C for 1 h per day. Animals were euthanized at 7 or 60 dpi and their hearts harvested for sectioning (Figure 4J). To learn if cardiomyocyte cell cycle entry is suppressed by Ruvbl2 overexpression, we immunostained cardiac sections from 7 dpi heat-shocked control and OE^hs^ fish for PCNA to mark cells undergoing DNA replication and Mef2 to identify cardiomyocyte nuclei. The proportion of PCNA+ cardiomyocytes in the border zone of OE^hs^ hearts was significantly reduced compared to controls (Figures 4K–M), demonstrating that Ruvbl2 suppresses cardiomyocyte proliferation during adult heart regeneration. To learn if overexpression of Ruvbl2 inhibits heart regeneration, we performed AFOG staining on control and OE^hs^ hearts at 60 dpi. Control animals that were injured, heat shocked, and analyzed in parallel achieved robust myocardial regeneration with minimal levels of scar tissue (Figure 4N). By contrast, the majority of OE^hs^ hearts showed deficiencies in muscle renewal that is evidenced by retention of collagen-rich scar tissue and failures to close the myocardial wall (Figure 4O). While decreased cardiomyocyte proliferation correlates with reduced regenerative capacity in OE^hs^ hearts, we cannot rule out non-myocardial defects that might contribute to scar formation as Ruvbl2 expression is ubiquitous following heat shock. Overall, these findings demonstrate that forced expression of Ruvbl2 during the regenerative window inhibits cardiomyocyte proliferation and induces scarring.

## Discussion

We demonstrate that Ruvbl2 suppresses cardiomyocyte proliferation during developmental heart growth and adult heart regeneration in zebrafish. This anti-proliferative activity is surprising given the well-established oncogenic role of Ruvbl2 in a variety of cancers including renal, liver, lung, stomach, pancreas, and blood (Mao and Houry, 2017). Outside of a pathogenic setting, Ruvbl2 has also been shown to promote hepatocyte proliferation and survival during liver regeneration in mice (Javary et al., 2021). Given its pro-proliferative properties in other organs, our finding that Ruvbl2 is a potent suppressor of cardiomyocyte proliferation in the zebrafish heart suggests that diverse downstream functions are employed based on cell type. As such, elucidating the molecular mechanisms by which Ruvbl2 controls cardiomyocyte proliferation will rely on tissue-specific approaches.

Ruvbl2 is known to repress gene transcription through at least two different mechanisms. First, Ruvbl2 physically interacts with sequence-specific transcription factors to directly suppress target gene expression (Gallant, 2007). Secondly, Ruvbl2 functions as a critical ATPase in well-established chromatin remodeling complexes to reposition nucleosomes and alter the chromatin landscape (Gallant, 2007), which in turn affects gene transcription. Whether either mechanism is at play in cardiomyocytes is not currently clear. Recently, the Tip60 complex, which contains Ruvbl2, was shown to suppress cardiomyocyte proliferation following coronary artery ligation in adult mice (Wang et al., 2021). Specifically, genetic depletion of Tip60 after myocardial infarction increased cardiomyocyte proliferation and reduced scarring. Based on our findings in zebrafish, it is tempting to speculate that Ruvbl2 is functioning with Tip60 to control myocardial proliferation in the context of developmental heart growth and regeneration. However, immunoprecipitation-mass spectrometry studies will be required to identify the array of proteins that physically associate with Ruvbl2 in cardiomyocytes. Moreover, cardiomyocyte-specific surveillance of chromatin architecture will be needed to identify critical loci that might be altered in the presence and absence of Ruvbl2.

Overall, our findings directly implicate Ruvbl2 in suppressing cardiomyocyte proliferation *in vivo*. While signals that promote cardiomyocyte proliferation tend to be more prevalently studied (Yin et al., 2021), our data highlight the importance of balancing signals that stimulate cardiomyocyte proliferation with those that restrict it to achieve optimal heart muscle growth and regrowth. Ultimately, these findings might serve as novel inroads for fine-tuning cardiomyocyte proliferation in the context of regenerative medicine and lead to new therapeutics that would benefit patients suffering a myocardial infarction.

## Data Availability

The original contributions presented in the study are included in the article/supplementary material. Further inquiries can be directed to the corresponding authors.
