# Supplementary figures and images for: Ruvbl2 Suppresses Cardiomyocyte Proliferation During Zebrafish Heart Development and Regeneration
Source: Front Cell Dev Biol. 2022 Feb 1;10:800594. doi: 10.3389/fcell.2022.800594 (PMC8844374; doi:10.3389/fcell.2022.800594)

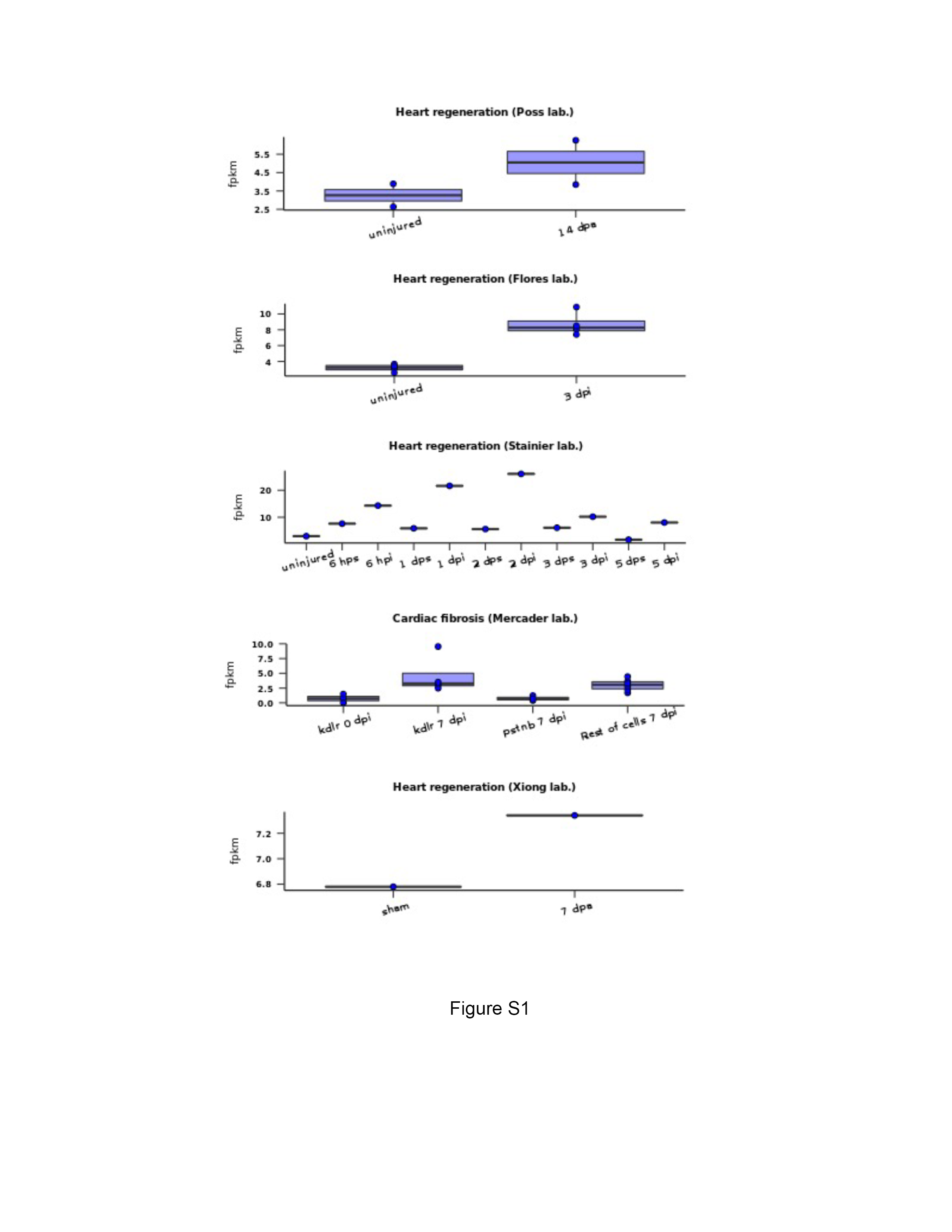

Supplement: Supplementary file 1 [file Image1.TIF]
